# Molecular Characterization of *Klebsiella pneumoniae* Carbapenemase (KPC)-Producing *Enterobacteriaceae* in Ontario, Canada, 2008-2011

**DOI:** 10.1371/journal.pone.0116421

**Published:** 2014-12-30

**Authors:** Nathalie Tijet, Prameet M. Sheth, Olga Lastovetska, Catherine Chung, Samir N. Patel, Roberto G. Melano

**Affiliations:** 1 Public Health Ontario Laboratories, Toronto, Ontario, Canada; 2 Department of Pathology and Molecular Medicine, Queen’s University, Kingston, Ontario, Canada; 3 Department of Laboratory Medicine and Pathobiology, University of Toronto, Toronto, Ontario, Canada; 4 Department of Microbiology, Mt Sinai Hospital, Toronto, Ontario, Canada; University Medical Center Groningen, Netherlands

## Abstract

Due to the lack of detailed reports of *Klebsiella pneumoniae* carbapenemase (KPC)-producing enterobacteria in Ontario, Canada, we perform a molecular characterization of KPC-producing *Enterobacteriaceae* submitted to the provincial reference laboratory from 2008 to 2011. Susceptibility profiles were accessed by E-test. Molecular types of isolates were determined by pulse-field gel electrophoresis (PFGE) and multilocus sequence typing. Screening of ß-lactamase genes was performed by multiplex PCR and alleles were identified by DNA sequencing. The genetic platform of *bla*_KPC_ gene was analyzed by PCR. Plasmid replicons were typed using PCR-based typing approach. KPC-plasmids were also evaluated by S1 nuclease-PFGE and Southern blot. Thirty unique clinical isolates (26 *Klebsiella pneumoniae*, 2 *Enterobacter cloacae*, 1 *Citrobacter freundii* and 1 *Raoultella ornithinolytica*) were identified as *bla*_KPC_ positive: 4 in 2008, 3 in 2009, 10 in 2010 and 13 in 2011. The majority exhibited resistance to carbapenems, cephalosporins and fluoroquinolones and two isolates were also resistant to colistin. The isolates harbored *bla*_KPC-2_ (n = 23) or *bla*_KPC-3_ (n = 7). *bla*_TEM-1_ (n = 27) was commonly detected and occasionally *bla*_OXA-1_ (n = 3) and *bla*_CTX-M-15_ (n = 1). As expected, all *K. pneumoniae* isolates carried *bla*_SHV-11_. *bla*_KPC_ genes were identified on Tn*4401a* (n = 20) or *b* (n = 10) isoforms, on plasmids of different sizes belonging to the incompatibility groups IncFIIA (n = 19), IncN (n = 3), IncI2 (n = 3), IncFrep (n = 2) and IncA/C (n = 1). The occurrence of KPC ß-lactamase in Ontario was mainly associated with the spread of the *K. pneumoniae* clone ST258.

## Introduction

*Klebsiella pneumoniae* carbapenemases (KPC) are serine ß-lactamases predominantly found in *Enterobacteriacae* (mainly in *K. pneumoniae*, but also in other species such as *Escherichia coli*, *Enterobacter* spp. or *Citrobacter freundii*) as well as in *Acinetobacter* spp. and *Pseudomonas aeruginosa* [1]–[3]. They confer resistance to all ß-lactams and most of the time are carried by multidrug-resistant clinical isolates [1]. Infections due to these microorganisms limit treatment options for patients and are associated with poorer outcomes, longer hospitalizations, and increased morbidity and mortality [1], [4]. Since the first KPC variant was detected in North Carolina, United States, in the late 1990s, KPC-producing bacteria have rapidly emerged worldwide [5], [6]. To date, 21 KPC variants have been described (KPC-2 to -22; http://www.lahey.org/Studies/, accessed on October, 2014). *bla*_KPC_ is a plasmid-carried gene harbored on a Tn*3*-like transposon, called Tn*4401*, a highly mobile genetic element [7]. However, its spread in bacterial populations is mainly due to a major clone of *K. pneumoniae*, defined by multilocus sequence typing (MLST) as sequence type (ST) 258. *K. pneumoniae* ST258 is largely responsible for KPC dissemination throughout North America and other parts of the world [8]. In Canada there are only two national surveillance studies that included KPC-producing enterobacteria data [9], [10]. In both of them, very few isolates were described (7 in reference 9, and 3 in reference 10, all recovered from provinces of Quebec and Ontario); interestingly, all the isolates where KPC-3 producers. In Ontario, the first *bla*_KPC_-harboring isolates were described in 2008 [11], [12]. Because there are not detailed molecular data of KPC-producing enterobacteria isolated in Ontario, Canada's most populous province (13.5 million, http://www.statcan.gc.ca/tables-tableaux/sum-som/l01/cst01/demo02a-eng.htm), the aim of this study was to perform the molecular characterization (molecular typing, identification of the genetic platforms and study of KPC plasmids) of KPC-producing *Enterobacteriaceae* submitted to the Public Health Ontario laboratories (PHOL) between 2008 and 2011.

## Materials and Methods

### Bacterial strains and antimicrobial susceptibility testing

All isolates recovered from clinical and screening specimens (one per patient) submitted to PHOL between January 2008 and December 2011, for confirmation of carbapenemase production, with MICs for meropenem or ertapenem of ≥2 µg/ml and ≥1 µg/ml, respectively, were included in this study. All these isolates were submitted with species identification and susceptibility profiles obtained by automated systems in each health care provider. Species identification was confirmed by biochemical assays, and, in the case of 1 *Raoultella* spp., also by 16S rRNA and *rpoB* sequencing analysis [13]. Carbapenemase activity in those isolates was detected using the modified Hodge test. The MICs for the KPC-positive isolates were determined using E-test (Biomerieux, Marcy L’Etoile, France) to several antimicrobials including ampicillin, cefoxitin, ceftazidime, cefotaxime, cefepime, ertapenem, imipenem, meropenem, amikacin, gentamicin, tobramycin, ciprofloxacin, tetracycline, tigecycline and colistin. Susceptibility results were interpreted according to the Clinical and Laboratory Standards Institute (CLSI) guidelines [14] except for colistin and tigecycline (susceptibility interpreted according to the European Committee on Antimicrobial Susceptibility Testing -EUCAST- guidelines, available at http://www.eucast.org/).

### Molecular methods

Multiplex-PCR based screening of the most commonly found ß-lactamase families (*bla*_TEM_, *bla*_SHV_, *bla*_OXA-1-like_, *bla*_CTX-M_ groups 1, 2 and 9, *bla*_VEB_, *bla*_PER_ and 6 groups of *bla*_AmpC_ genes) including carbapenemase genes (*bla*_KPC_, *bla*_OXA-48-like_, *bla*_IMP_, *bla*_VIM_, *bla*_NDM_, *bla*_GES_) was performed as previously described in all the isolates showing the carbapenems susceptibility criteria described above [15], [16]. To identify the alleles of ß-lactamases genes detected in the PCR screening, amplification of whole genes were performed using specific primers [17] and the PCR product sequenced using a 3130xl Genetic Analyzer (Life Technologies). Sequences were analyzed using Vector NTI Advance software (version 11.5.3; Life Technologies). Searches of sequences were performed with the BLAST program, available at the National Center for Biotechnology Information Web site (http://www.ncbi.nim.nih.gov/). Multiple-sequence alignments were performed with the ClustalX program, available at the European Bioinformatics Institute Web site (http://www.ebi.ac.uk/Tools/msa/clustalw2).

### MLST and pulsed-field gel electrophoresis (PFGE)

*K. pneumoniae* isolates were genotyped by MLST and PFGE. *K. pneumoniae* MLST was performed as described and the MLST database was used to assign allelic numbers and ST (available at http://www.pasteur.fr/recherche/genopole/PF8/mlst/Kpneumoniae.html) [18]. PFGE was performed using *Xba*I-digested genomic DNA as described [19]. The results were analyzed using the BioNumerics software (version 6.6; Applied Maths, Saint Martens-Latern, Belgium).

### Plasmid characterization

Plasmid replicons were typed using PCR-based typing approach as described by Carattoli et al. [20]. In order to define the presence of KPC-IncI2 plasmids, not detected using the PCR-based replicon typing but increasingly disseminated in New York and New Jersey, US states neighboring to Ontario, primers recently described by Chen et al were used [21]. The genetic surrounding of *bla*_KPC_ was studied by PCR mapping and sequencing [22]. Plasmid content and their estimated sizes were determined by S1 endonuclease-digested genomic DNA and PFGE (S1-PFGE) [23]. Plasmids carrying *bla*_KPC_ genes and their replicons were identified by S1-PFGE followed by Southern blot analysis using specific probes for *bla*_KPC_ and positive replicons (DIG Probe, Roche Diagnostics) [24].

## Results and Discussion

### Description of isolates

During the period studied 30 KPC-producing enterobacteria (4 isolated in 2008, 3 in 2009, 10 in 2010 and 13 in 2011), including the first one described in Ontario [11], were identified as *bla*_KPC_ positive at PHOL. These 30 isolates (26 *K. pneumoniae*, 2 *Enterobacter cloacae*, 1 *Raoultella ornithinolytica*, and 1 *C. freundii*) were submitted from 12 different health care providers within the province of Ontario. Majority of the isolates (n = 24) were submitted from the Greater Toronto Area which is the most populous region in Ontario. The median age of patients was 73 years old, with equal representation of both sexes. Most of the isolates were collected from urine/urinary catheters (n = 15, 50%) and rectal swabs (n = 9, 30%). Other sites of isolation included intra-peritoneal fluid, sputum, and peritoneal dialysis fluid.

### Antimicrobial susceptibility

Susceptibility profiles against 15 antimicrobials agents are listed in Table 1. As expected, the majority of the isolates exhibited resistance to most ß-lactams. Most of the isolates were also resistant to ciprofloxacin, tobramycin and amikacin. On the other hand, most of them were susceptible to gentamicin, tetracycline and colistin, and all were susceptible to tigecycline. Interestingly, the *R. ornithinolytica* isolate GN581/10 displayed resistance only to ampicillin, intermediate resistance to cefotaxime, ertapenem and imipenem, and susceptibility to all the other antibiotics tested. To our knowledge, there is only one publication describing KPC-producing *Raoultella* spp. [25]. In that study, one *R. ornithinolytica* resistant to all ß-lactam tested (including MICs >8 µg/ml to ertapenem, meropenem and imipenem) was characterized. *bla*_KPC_ gene promoter comparison was performed to relate the low level of ß-lactam resistance observed in isolate GN581/10 with the expression of KPC (see below in the ‘Characterization of *bla*_KPC_ genetic environment’ section).

**Table 1:** Susceptibility profiles of KPC-producing clinical isolates (N = 30).

		n (%) of isolates^a^			MIC (µg/ml)	
Antibiotics	Resistant	Intermediate	Susceptible	MIC_50_	MIC_90_	Range
Cefoxitin	28 (93.3)	1 (3.3)	1 (3.3)	48	≥256	6– ≥256
Ceftazidime	28 (93.3)	1 (3.3)	1 (3.3)	≥256	≥256	3– ≥256
Cefotaxime	29 (96.7)	1 (3.3)	0 (0)	32	≥256	2– ≥256
Cefepime	29 (96.7)	0 (0)	1 (3.3)	16	96	1.5– ≥256
Ertapenem	29 (96.7)	1 (3.3)	0 (0)	≥32	≥32	1– ≥32
Meropenem	24 (80)	3 (10)	3 (10)	6	≥32	0.75– ≥32
Imipenem	25 (83.3)	5 (16.7)	0 (0)	12	≥32	1.5– ≥32
Amikacin	21 (70)	2 (6.7)	7 (23.3)	48	96	1– ≥256
Gentamicin	8 (26.7)	6 (20)	16 (53.3)	3	16	0.19–96
Tobramycin	27 (90)	1 (3.3)	2 (6.7)	24	64	0.25–96
Ciprofloxacin	28 (93.3)	0 (0)	2 (6.7)	≥32	≥32	0.012– ≥32
Tetracycline	4 (13.3)	10 (33.3)	16 (53.3)	4	12	0.75– ≥256
Tigecycline	0 (0)	0 (0)	30 (100)	0.5	0.75	0.125–0.75
Colistin	2 (6.7)	NA	28 (93.3)	0.125	0.125	0.094– ≥32

a Susceptibility categories were defined according to CLSI breakpoints [14] except for colistin and tigecycline (EUCAST breakpoints; colistin: S, ≤2 µg/ml; R, >2 µg/ml; tigecycline: S, ≤1 µg/ml; R, >2 µg/ml).

### Molecular typing

**Figure 1:**
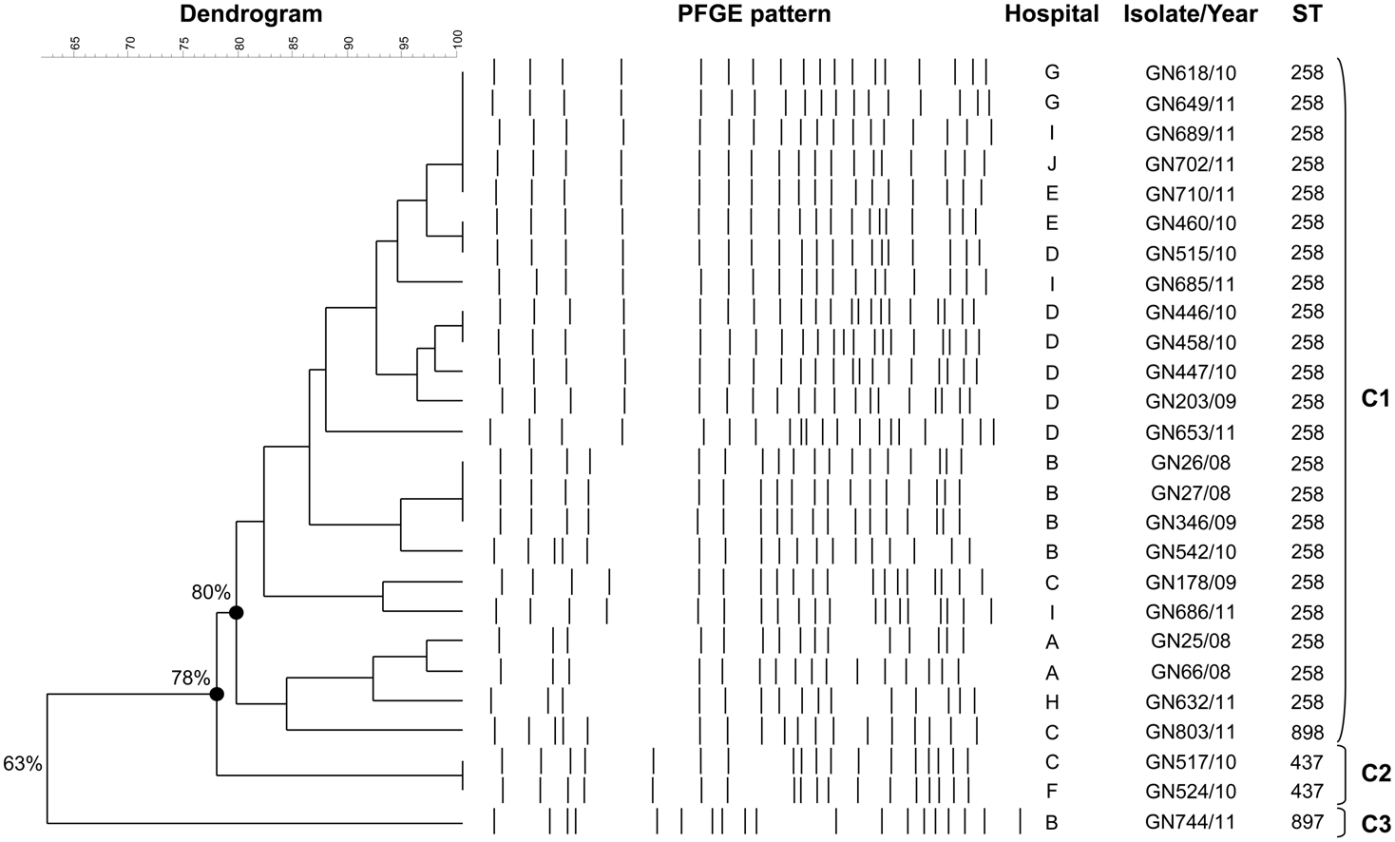
UPGMA dendrogram based on PFGE pattern of 26 *K. pneumoniae* isolates and their sequence type. Percentage of similarities is indicated in the branches of the dendrogram.

MLST analysis revealed that most of *K. pneumoniae* isolates (25 out of 26 isolates, 96.1%) belonged to a dominant clone ST258 or the closely related ST437 (a single locus variant of ST258) and ST898 (double locus variant of ST258, displaying one point mutation in the gene *infB* and three in the gene *tonB*) (Fig. 1). The first isolate detected in Ontario in 2008 (GN27/08 in this study) [11] was also typed as ST258. The remaining isolate was typed as ST897 (a single locus variant of ST15, displaying one point mutation in the gene *InfB*). *K. pneumoniae* ST258 clone has been recognized as the main KPC-disseminator in the US and worldwide [26]. Previous studies in Canada have showed that the few KPC-producing *K. pneumoniae* isolates described were typed mostly as ST258 or related [9], [10], [27], [28]. In a recent study, phylogenetic analysis of SNPs in the core genome of 85 *K. pneumoniae* ST258 isolates have showed that this clone is composed by 2 well defined lineages or clusters, mainly differentiated by a region of divergence that include the capsule polysaccharide biosynthesis island [29]. Based in these findings, more detailed studies are needed to determine what lineages the ST258 isolates from Ontario and the rest of the world actually belong to.

PFGE profiles showed the presence of a large cluster (C1) containing 23 isolates that are closely related (≥80% similarity) (Fig. 1). This cluster included the 22 ST258 isolates and one ST898. The results suggest transmission in 2 health care providers (hospital B, isolates GN26/08 and GN27/08 collected over two weeks in May 2008; hospital D, isolates GN446/10, GN447/10 and GN458/10 collected over three weeks in February 2010) (Fig. 1). Two minor PFGE types were also observed: cluster C2 included the two ST437 isolates, recovered in two different hospitals from rectal swabs from a woman and her son. This clone was KPC-2-producer, harboring the carbapenemase gene associated to Tn*4401*b on a 50 kb IncN plasmid (Table 2). This combination (ST437 carrying IncN *bla*_KPC-2_-plasmids) was previously found in isolates from Brazil [30]. Cluster C3 included the ST897 isolate, positive for *bla*_KPC-3_ gene detected on a 70 kb IncN plasmid. As a single-locus variant of ST15, ST897 belongs to CC292, a clonal complex that includes many internationally prevalent and multiresistant STs, also associated with dissemination of carbapenemases (e.g. STs 11, 14, 15 and 258)[31]–[35].

**Table 2:** Molecular characteristics of KPC-producing clinical isolates in Ontario, 2008/2011 (N = 30).

Isolate/year^a^	KPC variant	Tn*4401* isoform	S1-PFGE bands^b^	Replicon types found^c^	KPC-plasmid (estimated size, Kb)^c^	Other ß-lactamase detected
Kpn GN25/08	KPC-3	b	3	IncFrep, IncI2	IncFrep (120), IncI2(80)	TEM-1, SHV-11
Kpn GN26/08	KPC-2	a	4	IncA/C, IncFIIA	IncFIIA (100)	TEM-1, SHV-11
Kpn GN27/08	KPC-2	a	4	IncA/C, IncFIIA	IncFIIA (100)	TEM-1, SHV-11
Kpn GN66/08	KPC-3	b	2	IncI2	IncI2 (70)	TEM-1, SHV-11
Kpn GN178/09	KPC-3	a	2	IncFIIA	IncFIIA (170)	TEM-1, SHV-11
Kpn GN203/09	KPC-2	a	2	IncFIIA	IncFIIA (110)	TEM-1, SHV-11
Kpn GN346/09	KPC-2	a	3	IncA/C, IncFIIA	IncFIIA (190)	TEM-1, SHV-11
Kpn GN446/10	KPC-2	a	3	IncFIIA, IncI2	IncFIIA (80)	TEM-1, SHV-11
Kpn GN447/10	KPC-2	a	3	IncFIIA	IncFIIA (80)	TEM-1, SHV-11
Kpn GN458/10	KPC-2	a	3	IncFIIA	IncFIIA (80)	TEM-1, SHV-11
Kpn GN460/10	KPC-2	a	3	IncFIIA	IncFIIA (100)	TEM-1, SHV-11
Kpn GN515/10	KPC-2	a	3	IncFIIA	IncFIIA (100)	TEM-1, SHV-11
Kpn GN517/10	KPC-2	b	2	IncN, IncFIIA	IncN (50)	OXA-1, SHV-11
Kpn GN524/10	KPC-2	b	2	IncN, IncFIIA	IncN (50)	OXA-1, SHV-11
Kpn GN542/10	KPC-2	a	3	IncA/C, IncFIIA	IncFIIA (190)	TEM-1, SHV-11
Kpn GN618/10	KPC-2	a	3	IncFIIA	IncFIIA (100)	TEM-1, SHV-11
Ror GN581/10	KPC-2	b	5	IncFrep	IncFrep (70)	Neg
Kpn GN632/11	KPC-3	b	2	IncI2	IncI2 (80), untypeable (120)	TEM-1, SHV-11
Kpn GN649/11	KPC-2	a	3	IncFIIA	IncFIIA (100)	TEM-1, SHV-11
Kpn GN653/11	KPC-2	a	4	IncA/C, IncFIIA	IncFIIA (100)	TEM-1, SHV-11
Kpn GN685/11	KPC-2	a	3	IncFIIA	IncFIIA (100)	TEM-1, SHV-11
Kpn GN686/11	KPC-2	a	2	IncFIIA	Untypeable (80)	TEM-1, SHV-11
Kpn GN689/11	KPC-2	a	3	IncFIIA	IncFIIA (100)	TEM-1, SHV-11
Kpn GN702/11	KPC-2	a	3	IncFIIA	IncFIIA (100)	TEM-1, SHV-11
Kpn GN710/11	KPC-2	a	4	IncA/C, IncFIIA	IncFIIA (100)	TEM-1, SHV-11
Kpn GN744/11	KPC-3	b	3	IncN	IncN (70)	TEM-1, SHV-11
Kpn GN803/11	KPC-2	a	2	IncA/C, IncFIIA	IncFIIA (190)	TEM-1, SHV-11
Ecl GN743/11	KPC-3	b	3	IncHI2	Untypeable (120)	TEM-1
Ecl GN799/11	KPC-3	b	1	Untypeable	Untypeable (50)	TEM-1, CTX-M-15, OXA-1
Cfr GN801/11	KPC-2	b	3	IncA/C	IncA/C (180), untypeable (200)	TEM-1

a Kpn, *Klebsiella pneumoniae*; Ror, *Raoultella ornithinolytica*; Ecl, *Enterobacter cloacae*; Cfr, *Citrobacter freundii*.

b Number of bands detected in the S1-PFGE gels, representing one plasmid each.

c Clinical isolates were negative for all the 18 plasmid replicons tested using Carattoli et al approach [20] and their plasmids were defined as untypeable.

A low prevalence of carbapenemase-producing *Enterobacteriaceae* was found in the period 2009–10 at national level (0.02%) (9). In that study, ten carbapenemase producing *Enterobacteriaceae* were identified: two NDM-1-, one SME-2- and seven KPC-3-producers. These KPC-producers (most of them from Ontario and Quebec) included 4 *K. pneumoniae* and one *E. coli*, *K. oxytoca* and *S. marcescens*. Noteworthy, two outbreaks of KPC-3-producing enterobacteria other than *K. pneumoniae* were more recently described in Quebec [36], [37]. In our study, only 4 isolates were not *K. pneumoniae*; however, an increased detection of KPC-producers other than *K. pneumoniae*, including *E. cloacae*, *C. freundii*, *E. coli*, *Klebsiella oxytoca* and *Serratia marcescens* was noticed in Ontario in 2012–2013 (PHOL, data not shown). These findings, also described in other countries [22], [38], indicate that although the ST258 *K. pneumoniae* clone is the predominant KPC-disseminator, other enterobacterial species are increasingly facilitating the spread of KPC.

### ß-lactamase content and plasmid characterization

All the isolates harbored either the *bla*_KPC-2_ (n = 23; 76.7%) or *bla*_KPC-3_ (n = 7; 23.3%) genes (Table 2), the most frequent allelic variants described worldwide [27]. Except for *R. ornithinolytica* (only positive for *bla*_KPC_, see above), the isolates carried the broad-spectrum ß-lactamase genes *bla*_TEM-1_ (n = 27), *bla*_OXA-1_ (n = 3) and the extended-spectrum ß-lactamase gene *bla*_CTX-M-15_ (n = 1) (Table 2). As expected, all *K. pneumoniae* isolates (n = 26) carried the chromosomal *bla*_SHV-11_ gene (no other *bla*_SHV-_ variant was found in these isolates) (Table 2). These results indicate that the cephalosporinase and carbapenemase activity in the bacterial sample analyzed may be ascribed to KPC expression, as previously described [1].

Further analysis of plasmid replicon typing revealed that only five out of 18 plasmid replicon types screened by PCR were detected: IncFIIA (n = 22, 71%), IncA/C (n = 8, 26%), IncN (n = 3, 13%), IncFrep (n = 2, 6%) and IncHI2 (n = 1, 3%) (Table 2). Additionally, IncI2 replicon was also detected in 3 isolates. S1 endonuclease-digested DNA followed by PFGE and Southern blots using specific probes for these 6 positive replicons showed that most of the isolates (n = 29) contained 1 (n = 18) or 2 (n = 11) identifiable incompatibility plasmid group. If the number of bands in the S1-PFGE gels, representing one plasmid each, is compared to the identified replicon types, most of the isolates displayed untypeable plasmids using the current protocol (Table 2) [20]. New identified replicon types (e.g. IncI2, prevalent in New Jersey/New York area) [21] have to be included in this approach to reduce methodological limitations.

For the analysis of KPC-plasmids, Southern blot of S1-PFGE gels using a specific *bla*_KPC_ probe identified plasmids ranging from 50 to 200 Kb. The results from KPC- and replicon-hybridization analysis showed that KPC-plasmids belonged to the incompatibility groups IncFIIA (n = 19; plasmid size between 80 and 190 kb), IncN (n = 3; 50 to 70 kb), IncFrep (n = 2; 70 and 120 kb), IncI2 (n = 3; 70–80 kb) and IncA/C (n = 1; 180 kb), the most common plasmid families harboring carbapenemase genes (Table 2) [21], [39]. Five isolates contained untypeable KPC-plasmids (50 to 200 kb). All KPC-plasmids belonging to the IncI2 group were identified as pBK15692-like by PCR mapping, associated with KPC-3-harboring ST258 isolates, as described [21]. These three isolates (GN25/08, GN66/08 and GN632/11) were closely related by PFGE (>90% identity), but identified in 2 different hospitals (Fig. 1). IncI2 plasmids were recently found to be widely disseminated in New Jersey and New York hospitals in both *K. pneumoniae* and non-*K. pneumoniae* KPC-producing *Enterobacteriaceae* isolates, collected in the period 2007–2011 [21]. In our study, IncFIIA KPC-plasmids were prevalent and we scarcely detected IncI2 KPC-plasmids in the analyzed period (Table 2). A possible explanation of this different KPC-plasmid prevalence in the same period of time between neighbor regions (NJ/NY and Ontario) would be that KPC is endemic in the North East of the US (situation not observed in Ontario) and the consequent elevated chances of transmission of isolates harboring IncI2 KPC-plasmids between hospitals in the NJ/NY area. Additional studies are needed in both regions to decipher this different distribution.

KPC-plasmid replicon types were not identified in five isolates, including two *E. cloacae*, one *C. freundii* and two *K. pneumoniae*, three of which harbored *bla*_KPC-3_ gene. Two of these isolates, one *K. pneumoniae* (GN632/11) and one *C. freundii* (GN801/11), had two different KPC-plasmids: one untypeable (120 and 200 kb for GN632/11 and GN801/11 respectively) and the other belonging to IncI2 (GN632/11) or IncA/C (GN801/11) incompatibility group. All these results highlight the plasticity of KPC-plasmids belonging to the same incompatibility group, which would be prone to rearrangements. The results also suggest the important role played by Tn*4401* in the mobilization and dissemination of the *bla*_KPC_ genes between plasmids: two copies of the same Tn*4401b* isoform/*bla*_KPC_ allele located in 2 different plasmids were identified in 3 isolates (GN25/08, GN632/11 and GN801/11) (Table 2).

Previous studies have shown that the most prevalent KPC-plasmids belonged to IncFIIA, IncN and IncA/C incompatibility groups [9], [40]. Noteworthy, in our study we found IncFIIA plasmid only linked to *K. pneumoniae*. IncF plasmids are usually low copy number, narrow host range plasmids. The acquisition of multiple replicons has been described to be one way to expand their host range replication [41]. However, multi-replicon plasmids were not detected in any of the IncFIIA positive isolates studied here. This would be a reason of some unsuccessful attempts of transferring KPC-plasmids from *K. pneumoniae* to *E. coli* by conjugation (data not shown). This hypothesis is further supported by the fact that *bla*_KPC_ genes in enterobacterial species other than *K. pneumoniae* were not carried by IncFIIA plasmids (one IncA/C and one untypeable in *C. freundii*, IncFrep in *R. ornithinolytica*, or untypeable in the two *E. cloacae*). Similar results have been previously observed in a study from Argentina, where transconjugant *E. coli* strains harboring plasmids only from the IncL/M group or untypeable were obtained from clinical donors from species other than *K. pneumoniae* [22].

### Characterization of *bla*_KPC_ genetic environment

*bla*_KPC-_ genes were found on Tn*4401* in all isolates. Analysis of the region immediately upstream of *bla*_KPC-_ gene showed that 20 isolates (66.7%) carried it in the Tn*4401*a isoform and 10 (33.3%) in the Tn*4401*b isoform (Table 2). Tn*4401*a was located almost exclusively on IncFIIA plasmids (only one was untypeable), carrying *bla*_KPC-2_ (n = 19) or *bla*_KPC-3_ (n = 1), while Tn*4401*b was detected on plasmids belonging to the incompatibility groups IncN (n = 3, *bla*_KPC-2_ or *bla*_KPC-3_), IncFrep (n = 2, *bla*_KPC-2_ or *bla*_KPC-3_), IncA/C (n = 1, *bla*_KPC-2_), IncI2 (n = 3, *bla*_KPC-3_) or on untypeable plasmids (n = 4, *bla*_KPC-2_ or *bla*_KPC-3_) (Table 2). These results would suggest a more active dissemination of *bla*_KPC_ genes between plasmids when they are on Tn*4401b* isoform.

Particular attention was paid to the *bla*_KPC_ promoter in *R. ornithinolytica* isolate GN581/10 due to the low carbapenems MICs observed compared to previously described *Raoultella* spp. [25]. Our results suggest that even if isolate GN581/10 has a complete *bla*_KPC-2_ gene carried on a Tn*4401* isoform b, it is either only marginally expressed or not expressed. Analysis of the promoter region sequence immediately upstream of *bla*_KPC-2_ gene in isolate GN581/10 did not show differences with other Tn*4401*b analyzed in this study. Nonetheless, further studies are required to determine the expression of KPC ß-lactamase in this isolate. Also, since no other ß-lactamase gene was detected in this *R. ornithinolytica* isolate, its resistance profile would be, in part, consequence of the expression of the chromosomally encoded ORN-1, a class A ß-lactamase with strong penicillinase activity previously characterized [42].

### Concluding remarks

This is the most detailed molecular description of KPC-producing *Enterobacteriaceae* in Ontario, Canada. In the period 2008–2011, the occurrence of KPC-producing enterobacteria was associated with a *K. pneumoniae* dominant clone (ST258 or closely related) and a dominant incompatibility type plasmid (IncFIIA). However, diversity of plasmid sizes and genetic platform carrying *bla*_KPC_ was also observed, even among isolates with the same fingerprint pattern and ST. Although the number of isolates observed at PHOL was small in the period studied, a consistent increased number of KPC-producing isolates was observed since 2009 in Ontario, which highlights the importance of continued surveillance of this resistance mechanism.
